# Investigation of Night Pain in Geriatric Regional Degenerative Pain and Associated Conditions

**DOI:** 10.5152/eurasianjmed.2026.251085

**Published:** 2026-02-20

**Authors:** Bilgehan Kolutek Ay, Cem Zafer Yıldır

**Affiliations:** Department of Physical Medicine and Rehabilitation, Sütçü İmam University Faculty of Medicine Kahramanmaraş, Türkiye

**Keywords:** Geriatric patients, osteoarthritis, pain management, sleep disorders

## Abstract

**Background:**

Chronic and night pain are common challenges in the geriatric population, often complicating pain management. This study aimed to evaluate sleep quality, night pain frequency, and associated factors in regional degenerative pain among elderly patients to identify individuals at risk for night pain early.

**Methods:**

A total of 130 geriatric patients diagnosed with degenerative osteoarthritis and experiencing regional chronic pain were included. Demographic data, sleep quality, and bone mineral density were assessed. Patients were grouped based on the presence of night pain, and comparisons were made accordingly.

**Results:**

Night pain was reported by 95 patients (73%), and 76 participants (58%) had poor sleep quality. Those with night pain showed significantly worse sleep quality (*P* < .001). No significant differences were found between groups in terms of pain location, gender, or number of chronic conditions (*P* = .41, *P* = .368, *P* = .206). However, night pain was significantly more common among participants with low education levels or illiteracy (*P* = .006). Resistance to simple analgesics was also higher in the night pain group (*P* = .009). Bone mineral density values did not significantly differ between groups (*P* = .785, *P* = .398, *P* = .232).

**Conclusion:**

Early identification of geriatric patients at risk for night pain is essential for optimal pain management. Poor sleep quality, inadequate response to analgesics, and lower education levels were significantly associated with night pain. Proactive follow-up and individualized treatment approaches may improve outcomes in this vulnerable population.

**Main Points**

- Night pain is a condition influenced by multiple factors in the geriatric population.
- Even in the absence of inflammation or malignancy, regional degenerative problems can cause night pain.
- Night pain negatively affects sleep quality.
- Patients with night pain tend to respond less favorably to simple analgesics.
- Lower educational attainment, inadequate response to basic analgesic therapy, and increased daytime pain intensity may represent potential risk factors for the development of night pain.

## Introduction

Musculoskeletal problems in the geriatric population are closely associated with pain, limited mobility, reduced quality of life, and sleep disturbances. These problems may result not only from degenerative changes but also from complications of chronic conditions such as diabetic neuropathy, hemiplegic shoulder, or central pain syndromes.^1^

When discussing degenerative pain, osteoarthritis is the most commonly considered condition. Osteoarthritis can develop in synovial joints throughout the body and may lead to either acute or chronic pain. While degenerative pain can sometimes be mild and manageable, it can also become progressively severe and change in character if left untreated. Typically nociceptive in nature, degenerative pain may evolve into a nociplastic or neuropathic pattern over time. As the character of the pain changes, its responsiveness to simple analgesics diminishes, and it may even begin to occur during rest, especially at night, thereby disrupting sleep.^2^^,^^3^

Non-inflammatory, non-cancerous, and non-infectious night pain is a multifactorial issue in the elderly that significantly impairs sleep and quality of life.^4^ While the presence of night pain in patients with regional osteoarthritis has been debated in the literature as a factor influencing surgical decisions, its occurrence in patients not at the surgical stage highlights the importance of further investigation.^5^ Moreover, managing pain in patients with night pain is often challenging regardless of surgical indication. The persistence of night pain in some patients even after surgery further underscores the complexity of this issue. Therefore, early identification of individuals at risk for night pain is of great importance.

This study aims to examine the link between night pain and the quality of sleep and to determine the factors contributing to night pain among individuals aged 65 and above.

## Material and Methods

### Study Design

This study employed a cross-sectional observational design and was implemented over a 3-month period in the outpatient service of the Physical Medicine and Rehabilitation Department at a tertiary care center.

### Participants

The study initially evaluated 204 participants, each aged at least 65 years, who presented to the outpatient department with regional pain and received a diagnosis of degenerative joint disease. Following the diagnostic process, patients were excluded if they had inflammatory or infectious diseases, malignancy, neurological conditions such as stroke, multiple sclerosis, or dementia, cognitive impairment, known sleep disorders under medical treatment, entrapment neuropathies, peripheral neuropathy, or a history of trauma. Ultimately, the study included 130 participants who fulfilled the inclusion requirements. Their medical diagnoses comprised conditions such as spondylosis, listhesis, intervertebral disc herniation, spinal canal stenosis in vertebral segments, and degenerative ligament or tendon pathologies, including osteoarthritis, in joints like the knee, shoulder, and ankle. For patients with diagnoses in multiple regions, only the region associated with their primary complaint was included in the evaluation.

### Demographic Data Evaluation

For all patients included in the study, general demographic data were recorded, including age, sex, body mass index (BMI), level of education, duration of pain, and the number and type of accompanying chronic diseases.

### Pain Evaluation

Pain intensity was assessed using the Visual Analog Scale (VAS). Daytime pain was evaluated in all patients, and nighttime pain was additionally assessed in patients reporting nocturnal symptoms. The VAS consists of a 100-mm horizontal line, with the left end indicating “no pain” and the right end indicating “worst imaginable pain.” Patients were asked to mark the point on the line that best represented their pain intensity. The distance in millimeters from the “no pain” anchor to the marked point was recorded as the VAS score.

### Bone Mineral Density Evaluation

Bone mineral density (BMD) measurements were obtained from records within the previous 3 months. Measurements included femoral neck T-scores, total femur T-scores, and lumbar spine (L1-L4) T-scores. All eligible measurements were performed at the same hospital using the same dual-energy X-ray absorptiometry (DXA) device. For patients without available BMD data meeting these criteria, new assessments were performed using a DXA scanner (GE Healthcare, Lunar model 8548, USA).

### The Pittsburgh Sleep Quality Index Evaluation

Sleep quality was evaluated using the Pittsburgh Sleep Quality Index (PSQI). The PSQI was originally developed by Buysse et al and subsequently validated for reliability in the Turkish population by Ağargün et al. The index consists of 18 items grouped into 7 components assessing sleep quality and sleep disturbances over the preceding month. Each component is scored from 0 to 3, with higher scores indicating poorer sleep quality. The global PSQI score ranges from 0 to 21, with scores greater than 5 indicating poor overall sleep quality.

### Evaluation of the Relationship Between Sleep Quality and Pain

After completion of data collection, patients were categorized according to the presence or absence of nighttime pain. Comparative analyses were then conducted to evaluate the relationship between sleep quality, pain intensity, and BMD parameters.

### Ethical Approval

Approval for the research was granted by the Ethics Committee on Medical Research at Sütçü İmam University (Date: June 4, 2024 Protocol No: 167). In line with the Declaration of Helsinki, the study was carried out following established ethical guidelines, with written informed consent obtained from all individuals taking part.

### Statistical Analysis

Data analysis was performed using SPSS software version 15.0 (IBM Corp., Armonk, NY, USA). The normality of continuous variables was assessed with the Shapiro–Wilk test. Variables that followed a normal distribution are reported as means with standard deviations, while those that did not are summarized by medians and ranges (minimum to maximum). Categorical variables are presented as counts and percentages. To compare continuous variables between 2 independent groups, the independent samples *t*-test was used for normally distributed data, and the Mann–Whitney *U*-test was applied when data were not normally distributed. For parameters that showed significant differences between the 2 groups, receiver-operating characteristic (ROC) analysis was performed to determine the optimal cut-off point and to evaluate sensitivity and specificity. The area under the curve was calculated to assess test accuracy, and the optimal cut-off point was manually selected based on the highest sensitivity and specificity. The categorical data were analyzed using the chi-square test. Statistical significance was set at *P *≤ .05.

## Results

Within the sample of 130 patients, lumbar pain was the most frequently reported, representing 38% of all cases. Night pain was present in 95 of the 130 patients. All 13 patients with shoulder involvement reported night pain, and 6 of these patients also had a diagnosis of coronary artery disease (CAD) (Table 1). No meaningful differences in age, BMI, pain duration, or T-scores for L1-L4, total femur, and femoral neck were identified when comparing patients grouped by the presence or absence of night pain. Those experiencing night pain had markedly higher daytime VAS scores (*P* = .002), and their mean nighttime VAS score was calculated as 7.26 ± 7.31. Evaluation of categorical variables showed no statistically significant variation between the night pain and non-night pain groups in terms of gender, pain location, comorbidity count, or diagnoses of hypertension, diabetes mellitus, or CAD. PSQI assessments indicated that poor sleep quality was more prevalent among patients with night pain, with statistical significance (*P* < .001). When patients were compared based on educational status, night pain was more common among those who were illiterate and had no formal education (*P* = .006). A positive response to simple analgesics was more frequently observed in the group without night pain (*P* = .009). In this study, the most common comorbidity accompanying night pain was hypertension (n = 57) (Table 2). Receiver-operating characteristic analysis for the daytime VAS score determined an optimal cut-off value of 5.50 (Table 3).

## Discussion

This study evaluated factors associated with regional degenerative conditions and night pain in a geriatric patient population.

Previous research indicates that chronic pain among older adults is most frequently attributed to musculoskeletal conditions, particularly involving the lumbar region and various joints, and the distribution of pain regions as well as the higher prevalence observed among female patients in the study are consistent with these previous findings.^1^

Degenerative musculoskeletal pain in the elderly has been shown to be associated with a variety of problems, including sleep disturbances, increased risk of falls, and depressive symptoms.^5^ In this research, the presence of night pain was associated with a significant decline in sleep quality. Numerous studies have investigated the association between chronic pain and disturbances in sleep patterns. Studies have demonstrated that chronic pain can lead to sleep disorders, and conversely, individuals with sleep disturbances may experience more chronic pain. Evidence indicates that sleep and pain may influence each other bidirectionally. Mechanistic explanations for this link include the involvement of opioid and monoaminergic pathways, the endocannabinoid system, hypothalamic-pituitary-adrenal axis activity, and melatonin control.^6^^,^^7^ When reviewing studies on chronic pain, it becomes evident that sociocultural factors and educational level play important roles in pain perception and management.^8^ The study found a negative relationship between education level and night pain: illiterate individuals were more likely to experience night pain. Similarly, prior studies have indicated that lower educational attainment correlates with an increased occurrence of chronic pain among older individuals. In one report, 48% of seniors experiencing chronic pain had no formal education.^1^

In this study, daytime pain intensity was found to be higher in patients with night pain. The literature suggests that individuals who experience pain during sleep are also more likely to report daytime pain, especially following activities such as grocery shopping, long walks, or carrying objects. These daytime activities can increase overall pain intensity and contribute to the occurrence of night pain.^9^^,^^10^For the elderly, initial analgesic strategies commonly involve the use of paracetamol and NSAIDs, both of which tend to be effective in controlling nociceptive pain. However, when nociceptive pain remains untreated for extended periods, it may evolve into a neuropathic or nociplastic type of pain, due to neuroplastic changes at the spinal and central nervous system levels. As the nature of pain shifts, managing regional pain often requires medications beyond paracetamol and NSAIDs.^1^^,^^3^^,^^4^^,^^5^^,^^11^

An important outcome of this study was that patients reporting night pain responded less effectively to NSAIDs. It is believed that this may be due to the aforementioned changes in pain processing. Pain that persists into the night, occurs at rest, and disrupts sleep may suggest an altered pain mechanism. This phenomenon should be carefully considered in the geriatric population. Prolonged use of NSAIDs in elderly patients who are unresponsive to these medications may lead to adverse effects such as gastrointestinal complications and impaired renal function, without offering meaningful pain relief. Instead, tailoring treatment to the specific nature of the pain may improve outcomes and is especially important in the geriatric population, where polypharmacy is already common.

The findings indicated that the number of chronic comorbidities did not differ significantly between patients experiencing night pain and those free of it. However, earlier research has indicated that the number of chronic illnesses may be linked to the occurrence of chronic pain in the elderly population.^4^^,^^5^ It was hypothesized that conditions linked to chronic pain may also serve as risk factors for night pain. Nevertheless, the scarcity of research dedicated to night pain underscores the importance of conducting additional studies on the subject.

In this study, hypertension was found to be the most common comorbid chronic disease, present in 57% (n = 72) of patients. Studies conducted worldwide and in Türkiye have shown that hypertension is the most frequently seen chronic disease in the geriatric population. The findings are consistent with the literature in this regard. According to the Turkey Hypertension Prevalence Study, the prevalence of hypertension in individuals over 60 years old reaches up to 67%.^12^ The lower rate in the study (57%) may be attributed to socio-economic and cultural differences in the region where the study was conducted, the generally low education levels, inclusion criteria selecting patients with specific diseases, and the fact that Kahramanmaraş, where the study took place, was affected by the 2023 earthquake, possibly causing underdiagnosis. Although hypertension alone is not typically considered a direct cause of chronic pain, studies have indicated its association with chronic low back pain, increased pain severity in knee osteoarthritis, and a higher frequency and intensity of general chronic pain in hypertensive patients.^13^^,^^14^ Additionally, it has been observed in a previous study that angiotensin-converting enzyme inhibitors, a commonly used class of antihypertensive drugs, may influence pain catastrophizing and are potentially associated with the development of Complex Regional Pain Syndrome.^15^ Even though no statistically significant difference was detected between the groups for hypertension and night pain in the study, the possible association between hypertension and pain should not be overlooked. The second most frequent chronic disease accompanying the patients was diabetes mellitus at 28%. Research indicates that diabetes is related to osteoarthritis and chronic pain, and when left uncontrolled, it can contribute to neuropathy, thereby worsening osteoarthritic pain.^16^^-^^18^

One notable result in the study was that all 13 patients with shoulder pathology reported night pain, and 6 of them had CAD. Literature indicates that CAD is a risk factor for shoulder pathologies, and night pain and sleep disturbances are commonly seen in patients with shoulder disorders. Previous studies have demonstrated that night pain is a characteristic feature of rotator cuff pathologies.^19^^,^^20^

Osteoporosis is a significant cause of morbidity in individuals over 65 years old. Evidence suggests that osteoporosis contributes to greater occurrence and severity of chronic pain, and its concurrence with night pain could be linked to non-radiographically detectable fractures.^21^^,^^22^No relationship was found between night pain and T-scores in the participants of the study. Nonetheless, findings from 1 investigation suggested a possible relationship between night pain and tibial BMD among patients with knee osteoarthritis.^23^ The fact that BMD measurements in the study were taken from lumbar and femoral regions, while the study included multiple osteoarthritis sites, may have prevented establishing this relationship.

### Limitations of the Study

The main limitations of this study include its observational and cross-sectional design, which restricts the ability to establish causal relationships between nocturnal pain and sleep quality. Conducted at a single tertiary care hospital’s outpatient clinic, the findings may have limited generalizability. The relatively small sample size of 130 participants may reduce the statistical power, especially for subgroup analyses. The lack of standardization in NSAID therapy, due to variations in active compounds and treatment durations among patients, represents another limitation of the study. Bone mineral density measurements were limited to the lumbar spine and femur regions, potentially overlooking associations in other osteoarthritic sites. Additionally, some relevant clinical and socio-demographic factors, such as psychological status, pain management details, and physical activity levels, were not comprehensively assessed.

## Conclusion

This study highlights that night pain is a prevalent and significant problem among the geriatric population with regional degenerative musculoskeletal conditions. Night pain is closely linked with poorer sleep quality, higher daytime pain intensity, and lower educational levels. Additionally, resistance to simple analgesics is more common in patients experiencing night pain, suggesting a possible change in pain mechanisms over time. Even though no meaningful link was found between night pain and BMD or the number of chronic conditions, hypertension was the most common comorbidity in this population. The findings point to the necessity of early detection and personalized management of night pain among older adults, with attention to clinical as well as socio-demographic considerations. Clinicians should be aware that standard analgesics may be insufficient for some patients, necessitating more comprehensive pain management strategies. Prospective investigations in the future are required to better understand the mechanisms behind night pain and to identify optimal therapeutic approaches for geriatric populations.

## Figures and Tables

**Table 1.** Pain Distribution of Patients

**Region**	**With Night Pain, n (%)**	**Without Night Pain, n (%)**	**Total, n**
Lumbar	38 (77.6)	11 (22.4)	49
Knee	29 (65.9)	15 (34.1)	44
Neck	5 (55.6)	4 (44.4)	9
Shoulder	13 (100.0)	0 (0.0)	13
Hip	4 (80.0)	1 (20.0)	5
Elbow-hand-wrist	3 (60.0)	2 (40.0)	5
Foot-ankle	2 (66.7)	1 (33.3)	3
Thoracic	1 (50.0)	1 (50.0)	2
Total	95 (73.1)	35 (26.9)	130

**Table 2.** Comparison of Parameters According to Night Pain

**Parameters**		**Without Night Pain** **(n = 35)**	**With Night Pain** **(n = 95)**	***P***
Gender	Male	14 (40.0%)	30 (31.6%)	.368
	Female	21 (60.%)	65 (68.4%)	
Age (year)		71.00 (65.00-85.00)	70.00 (65.00-93.00)	.634
Pain duration (month)		5.00 (1.00-120.00)	6.00 (1.00-120.00)	.281
BMI (kg/m^2^)		28.22 ± 4.33	29.64 ± 4.75	.124
Daytime VAS (cm)		6.00 (3.00-8.00)	7.00 (4.00-8.00)	**.002**
Night VAS (cm)			7.26 ± 7.31	
Educational status	Illiterate	11 (31.4%)	61 (64.2%)	**.006**
	Literate	8 (22.9%)	16 (16.8%)	
	Primary	11 (31.4%)	13 (13.7%)	
	High	5 (14.3%)	5 (5.3%)	
Pain region	Lomber	11 (31.4%)	38 (40.0%)	.412
	Knee	15 (42.9%)	29 (30.5%)	
	Other	9 (25.7%)	28 (29.5%)	
PSQI	Poor Sleep	3 (8.6%)	73 (76.8%)	**<.001**
	Good Sleep	32 (91.4%)	22 (23.2%)	
NSAIDs’ response	No	8 (22.9%)	46 (48.4%)	**.009**
	Yes	27 (77.1%)	49 (51.6%)	
Number of chronic diseases	No	16 (45.7%)	28 (29.5%)	.206
	1	14 (40.0%)	40 (42.1%)	
	2	5 (14.3%)	26 (27.4%)	
	3	0 (0.0%)	1 (1.1%)	
Hypertension	No	20 (57.1%)	38 (40.0%)	.081
	Yes	15 (42.9%)	57 (60.0%)	
Diabetes mellitus	No	28 (80.0%)	65 (68.4%)	.194
	Yes	7 (20.0%)	30 (31.6%)	
Coronary artery disease	No	33 (94.3%)	89 (93.7%)	.899
	Yes	2 (5.7%)	6 (6.3%)	
Lomber 1-4 T-scores		−0.93 ± 1.77	−0.84 ± 1.57	.785
Femur total T-scores		−1.17 ± 1.22	−1.37 ± 0.93	.398
Femur neck T-scores		−1.70 ± 1.26	−1.98 ± 0.89	.231

BMI, body mass index; NSAIDs, non-steroidal -antiinflammatory drugs; PSQI, Pittsburg Sleep Quality Index; VAS, Visual Analog Scale. Bold values indicate statistically significant results (p < 0.05).

**Table 3.** Receiver-Operating Characteristic (ROC) Analysis of Daytime Visual Analog Scale

**Parameter**	**AUC**	**Cut-off Value**	**Sensitivity ** **(%)**	**Specificity ** **(%)**	**Asymptotic 95% ** **CI**		*P*
					**Lower ** **Bound**	**Upper ** **Bound**	
Daytime VAS (cm)	0.671	5.50	81.1	48.6	0.559	0.783	**.003**

AUC, area under the curve; VAS, Visual Analog Scale. Bold value indicate statistically significant results (p < 0.05).

## Data Availability

The data that support the findings of this study are available on request from the corresponding author.
